# Sexual compulsion – Relationship with sex, attachment and sexual orientation

**DOI:** 10.1556/JBA.4.2015.1.6

**Published:** 2015-03-18

**Authors:** AVIV WEINSTEIN, LICHEN KATZ, HILA EBERHARDT, KOBY COHEN, MICHEL LEJOYEUX

**Affiliations:** ^1^Department of Behavioral Science, University of ArielArielIsrael; ^1^Department of Behavioral Science, University of ArielArielIsrael; ^1^Department of Behavioral Science, University of ArielArielIsrael; ^1^Department of Behavioral Science, University of ArielArielIsrael; ^2^Department of Psychiatry, Paris 7 University and Hospital Bichat Claude Bernard, AP-HP and Maison Blanche HospitalParisFrance; ^1^Department of Behavioral Science, University of ArielArielIsrael; ^2^Department of Psychiatry, Paris 7 University and Hospital Bichat Claude Bernard, AP-HP and Maison Blanche HospitalParisFrance

**Keywords:** sexual compulsivity, attachment, sex differences, homosexuality

## Abstract

**Background and aims:**

Sexual addiction, also known as hypersexual disorder, is associated with serious psychosocial problems for many people.

**Methods:**

This study used questionnaires to investigate the effects of gender, sexual orientation and attachment (avoidance and anxiety) on sexual compulsion among 100 heterosexual and homosexual men and women.

**Results:**

A positive correlation was found between anxious attachment and sexual compulsivity (*r* = 0.46; *p* < 0.01) and a positive correlation between avoidant attachment and sexual compulsivity (*r *= 0.39; *p* ≤ 0.01) in all participants. Secondly, an analysis of covariance showed a gender by sexual orientation interaction effect [*F*(1, 103) = 6.39, *p* < 0.01] but no attachment effect on sexual compulsivity. A follow-up comparison showed that lesbian women had higher rates of sexual compulsivity than heterosexual women [*t* (2, 50) = 5.08, *p* < 0.001] whereas there was non-significant difference in sexual compulsivity between homosexual and heterosexual men [*t* (2, 50) = 1.30, *p* = N.S.].

**Discussion:**

The results provide preliminary evidence for an association between attachment and sexual compulsivity and the effects of gender and sexual orientation on sexual compulsivity.

## INTRODUCTION

Sexual addiction, which is also known as hypersexual disorder, has been associated with serious psychosocial problems for many people although it has not been recognized as a disorder that merits inclusion in the DSM (Quadland, 1985) – see Karila et al. (2014) for review. Originally, Carnes (1983) published a book titled *Out of the shadows: Understanding sexual addiction,* which has raised interest in the area and facilitated a discussion on the best way to define and diagnose the disorder. Despite different views about pathological characteristics of sexual addiction there is an agreement that this is a progressive relapsing condition which does not merely refer to a pathological diagnosis of sexual lifestyle that is socially deviant (Edger, 2010).

Sexual addiction involves compulsive behaviors such as constantly seeking new sexual partners, having frequent sexual encounters, engaging in compulsive masturbation and frequently using pornography. Despite efforts to reduce or stop excessive sexual behaviors individuals find it difficult to stop and they engage in risky sexual activities, pay for sexual services and resist behavioral changes to avert HIV risk (Carnes, 1991; Coleman-Kennedy & Pendley, 2002; Coleman, Raymond & McBean, 2003; Kalichman & Rompa, 1995). Sexual compulsivity has been associated with the number of unprotected vaginal sex acts with female sexual workers, lower self-efficacy for condom use, greater use of illicit drugs, and more financial need (Semple et al., 2010).

Cognitive and emotional symptoms include obsessive thoughts of sex, feelings of guilt about excessive sexual behavior, the desire to escape from or suppress unpleasant emotions, loneliness, boredom, low self-esteem, shame, secrecy regarding sexual behaviors, rationalization about the continuation of sexual behaviors, indifference toward a regular sexual partner, a preference for anonymous sex, a tendency to disconnect intimacy from sex, and an absence of control in many aspects of life (Carnes, 2000, 2001; Carnes & Schneider, 2000; Coleman et al., 2003; Coleman-Kennedy & Pendley, 2002). Finally, some studies find that sexual addiction is associated with or in response to dysphoric affects (Black, Kehrberg, Flumerfelt & Schlosser, 1997; Raymond, Coleman & Miner, 2003; Reid, 2007; Reid, Carpenter, Spackman & Willes, 2008; Reid & Carpenter, 2009) or stressful life events (Miner et al., 2007).

Attachment theory (Bowlby, 1979, 1982) argued that early attachment experiences affect personal and social life, professional relationships, dealing with stress, mental and physical health and cognitive development. According to recent developments in attachment theory, those who developed a safe attachment style which is not anxious or avoidant during infancy can form healthy relationships in adolescence and adulthood and handle life problems (Uytun, Oztop, Esel & Mdusunen, 2013). Individuals with secure attachment are expected to have low chances of becoming addicted to sex since they regulate and limit their sexual activity more than those with insecure attachment (Zapf, Greiner & Carroll, 2008). Furthermore, individuals who are addicted to sex are looking for sexual activity without the need for emotional relationships and they are more likely to be characterized by avoidant or anxious attachment (Gentzler & Kerns, 2004).

Gay men are diverse with respect to the sexual behaviors they both desire and enact (Moskowitz & Roloff, 2010; Sanderson, 1994). Moreover, gay men differ from other groups in their sexual behavior. Research shows that, on average, gay men have more partners, engage in more risky sexual behavior, and are more likely to seek sexual sensation than other groups, such as heterosexual men, women and lesbians (Bailey, Gaulin, Agyei & Gladue, 1994; Ekstrand, Stall, Paul, Osmond & Coates, 1999; Thompson, Yager & Martin, 1993). But among homosexual men there is variability in the propensity to engage in compulsive unprotected sex. Meyer and Dean (1995) have reported that about 6% of their 149 young New York City gay men (aged 18–24 years) engaged in very high risk behavior, defined as unprotected receptive anal intercourse with multiple partners. It appears that very high risk takers are qualitatively different from other risk takers: they reported more mental health problems, including more drug use and higher levels of internalized homophobia and AIDS-related traumatic stress response. Furthermore, there are moderators of sexual behavior among gay men such as being in monogamous relationships. Also sexual health and sexual health behaviors for example sexually transmitted diseases (STDs) were most influential over the enactment of sexual behavior or desires (Moskowitz & Roloff, 2010).

Few studies investigated sexual compulsivity among heterosexual and homosexual men. Furthermore, to the best of our knowledge, the relationships between compulsive sexual behavior and attachment and sexual preference or orientation have not been investigated before. We have therefore investigated sexual compulsivity and attachment style among populations of heterosexual and homosexual men and women. We hypothesized that secure attachment would be associated with lower rates of sex compulsion. Secondly, that homosexual men and women would show higher levels of sexual compulsivity than heterosexual men and women. Thirdly, we hypothesized that attachment style might mediate between sexual orientation and sexual compulsion.

## PROCEDURE

### Participants

The participants of this study were recruited by research assistants at the Psychology Department of the University from forums on the Internet and social media sites that are used by the general public and the homosexual and lesbian community. The enrollment target was 120 participants and 104 were recruited. The recruitment lasted for three months. A hundred men and women over 18 years old participated in this study. See Table 1 for demographic characteristics in all participants.

**Table 1.** Demographic characteristics of all participants

	Males						Females					
	Heterosexual			Homosexual			Heterosexual			Homosexual		
	N	M	S.D.	N	M	S.D.	N	M	S.D.	N	M	S.D.
Age	26	7.83	4.97	26	26.17	3.13	26	29.58	5.9	26	24.5	1.86

Education (%)												
<12 years	4			0			0			8		
High-school	62			50			65			23		
Bachelor degree	30			50			31			42		
Master’s degree	4						4			23		
PhD	0									4		

Personal status (%)												
Single	84			92			84			81		
Separated/divorced	0			0			0			8		
Single	16			8			16			11		

Country of birth (%)												
Israel	92			92			92			92		
Overseas	8			8			8			8		

### Measures

Demographic questionnaire including details about sex, age, education, employment and sexual preferences.

Sexual Compulsivity Scale (SCS) by Kalichman and Rompa (1995) has 10 questions on compulsive sexual behavior, excessive sexual activity and compulsive sexual thoughts. The Sexual Compulsivity Scale was developed to assess tendencies toward sexual preoccupation and hyper-sexuality. Items were initially derived from self-descriptions of persons who self-identify as having a ‘sexual addiction’. The self-descriptors were taken from a brochure for a sexual addictions self-help group. The scale has been able to predict rates of sexual behaviors, numbers of sexual partners, practice of a variety of sexual behaviors, and histories of sexually transmitted diseases. Items were responded to on 4-point scales ranging from 1 *(very much like me)* to 4 *(very much not like me).* The scale is internally consistent with Alpha coefficients that range between .85 and .91 and in our study the scale had Cronbach internal validity of α = 0.94.

Experience in Close Relationship Scale (ECR) by Brennan, Clark and Shaver (1998) that was validated in Israel by Mikulincer and Florian (2000). The questionnaire has 36 questions divided equally into avoidance of intimacy and attachment and anxious attachment that is related to abandonment and separation anxiety. There are 4 combinations of attachment: secure attachment is indicated by low avoidance measures and low anxiety measures, avoidant attachment which is indicated by low anxiety measures, anxious attachment which is indicated by low avoidance measures and avoidant-anxious attachment which is indicated by high anxiety and high avoidance measures. Ratings are from 1 “strongly disagree” to 7 “agree very much”. In our study the part of the questionnaire on avoidance attachment had a Cronbach internal validity of α = 0.90 and the part on anxious attachment had a Cronbach internal validity of α = 0.87.

### Ethics

The study was approved by the Institutional Review Board (IRB-Helsinki committee) of the University. All participants gave informed consent to the study.

## RESULTS

### First hypothesis

The association between avoidance attachment, anxious attachment and sexual compulsivity was tested by a Pearson correlation analysis which showed a positive correlation between anxious attachment and sexual compulsivity (*r* = 0.46; *p* < 0.01) and a positive correlation between avoidant attachment and sexual compulsivity (*r* = 0.39; *p* ≤ 0.01). The results support our first hypothesis of an association between avoidant and anxious attachment and sexual compulsivity.

### Second hypothesis

The effects of gender and sexual orientation on ratings of sexual compulsivity were tested by an analysis of co-variance (ANCOVA) on the effects of gender and sexual orientation on ratings of sexual compulsivity using anxiety and avoidance measures of attachment and sexual orientation and gender as covariates. The results showed a non-significant gender effect [*F*(1, 103) = 1.74, *p* = N.S.], a non-significant sexual orientation effect [*F*(1, 103) = 0.85, *p* = N.S.], a non-significant attachment effect [*F*(1, 103) = 0.94; *p* = N.S.] and a significant gender by sexual orientation interaction [*F*(1, 103) = 6.39, *p* < 0.01].

A post-hoc analysis showed that lesbian women had higher ratings of sexual compulsivity than heterosexual women [*t*(2, 50) = 5.17, *p* < 0.001] and non-significant differences in sexual compulsivity between homosexual and heterosexual men [*t*(2, 50) = 1.22, *p* = N.S.]. A comparison of anxious attachment between lesbian and heterosexual women showed a significantly higher rating of anxious attachment in lesbian women than heterosexual women [*t*(1, 51) = 3.26, *p* < 0.001].

The results partially support the second hypothesis by indicating that lesbian women had higher ratings of sexual compulsivity than heterosexual women.

### The third hypothesis

Of differences in attachment between homosexual and heterosexual men and women was refuted due to the lack of attachment effect or interaction between attachment and sexual orientation in the ANCOVA.

Table 2 shows means and S.D. of ratings of sexual compulsivity, anxious and avoidant attachment in all participants.

**Table 2.** Means and S.D. of ratings of sexual compulsivity, anxious and avoidant attachment in all participants

	Heterosexual			Homosexual			Overall			*t*-test
Sexual compulsivity	N	M	S.D.	N	M	S.D.	N	M	S.D.	
Men	26	3.99	1.51	26	4.62	1.91	52	4.3	2.50	n.s
Women	26	3	0.94	26	3.5	0.53	52	4.2	3.15	5.17**
Overall	52	3.5	0.53	52	5	0.84	104	4.25	2.65	

Anxious attachment										
Men	26	6.97	1.97	26	9.4	2.62	52	8.18	1.16	n.s
Women	26	7.68	2.48	26	9.63	1.69	52	8.65	0.99	3.26**
Overall	52	7.3	1.01	52	8.5	0.91	104	8.42	1.3	

Avoidant attachment										
Men	26	3.45	0.74	26	3.78	1.13	52	3.61	0.95	n.s
Women	26	3.27	0.97	26	3.12	0.89	52	3.20	0.92	n.s
Overall	52	3.36	0.86	52	3.45	1.06	104	3.40	0.93	

** *p *< 0.001										

**Fig. 1.**
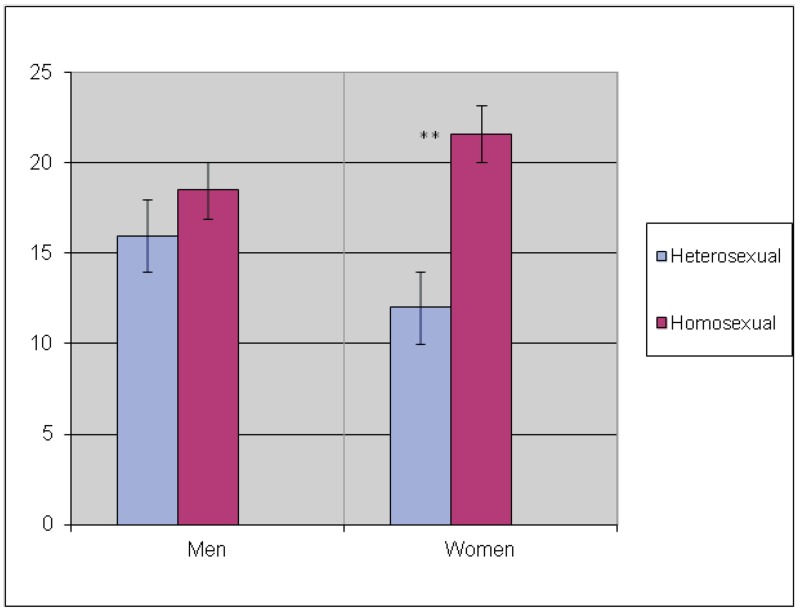
Mean sexual compulsivity scale

Figure 1 shows means of sexual compulsivity among homosexual and heterosexual men and women.

## DISCUSSION

This study showed a positive association between sexual compulsivity and anxious and avoidant attachment styles in all participants. These findings seem to suggest that high rates of sexual compulsivity are associated with difficulty in forming secure attachment in adult life. This evidence is compatible with attachment theory that postulates that difficulties in forming secure attachment with others are associated with problems in intimacy (Bowlby, 1988; Schwartz & Southern, 1999). According to Freeburg and van Winkle (2011) real persons cannot live up to the idealistic imaginings in virtual reality that a sexually compulsive person is seeking. The sexually compulsive persons yearn for close attachments, but their expectant models prevent any form of sustained intimacy (Griffin-Shelley, Benjain & Benjamin, 1995; Leedes, 2001; Leiblum & Rosen, 1988, 1989; Schore, 2001; Schwartz, 1996). Sexual addicts compensate for their inability to form close attachments by fantasizing about unattainable and unrealistic surrogates (Leedes, 2001; Schore, 2001; Schwartz, 1996; Zapf et al., 2008). Accordingly, individuals with anxious insecure attachment tend to be more vulnerable and seek sex as a source for comfort without a need for emotional intimacy (Zapf et al., 2008). It is plausible that sexual activity without commitment may also ease fears of separation and abandonment and therefore are favorable to the anxious types. The clinical evidence shows that those with avoidant attachment also seek sexual relationships without emotional commitment.

Secondly, lesbian women showed higher rates of sexual compulsivity than heterosexual women. This is a finding that to the best of our knowledge has not been shown before and it should be investigated further. The lack of difference in sexual compulsivity between homosexual and heterosexual men is also surprising in view of previous evidence for higher rates of sexual sensation seeking and sexual activity among this population (Moskowitz & Roloff, 2010). There is also evidence that homosexual men have more partners, engage in more risky sexual behavior, and are more likely to seek sexual sensation than other groups, such as heterosexual men, women and lesbians (Bailey et al., 1994; Ekstrand et al., 1999; Thompson et al., 1993). It is plausible that the sexual compulsive scale measured sexual preoccupation and hyper-sexuality which are different constructs than risk-taking behavior and sexual sensations that were measured in the previously reported studies. There is a need for a high reliability and validity tool for assessing sexual compulsivity and addiction.

Third, the study showed no conclusive evidence that homosexual men and lesbian women had higher rates of anxious or avoidant attachment than heterosexual participants. Although the post-hoc analysis found that lesbian women had higher anxious attachment scores than heterosexual women, this finding should be qualified by the lack of overall attachment effect or attachment by sexual orientation interaction in the ANCOVA. There are relatively few studies that looked into the relationship between attachment and sexual orientation. Ridge and Feeney (1998) have assessed attachment style, working models of attachment, early relationships with parents and relationship history, status and functioning among 70 homosexual and 100 lesbian and a comparison group of heterosexual men and women and found that relative frequencies of attachment styles were similar for homosexual and heterosexual samples. Overall, insecure attachment may not be over-represented in gay and lesbian samples, but insecurity was associated with less relationship satisfaction and with problems related to the disclosure of sexual orientation.

## LIMITATIONS

This study had a small sample size hence it is premature to extrapolate from its findings to sexual compulsivity and attachment style in the population of heterosexual and homosexual men and women. Secondly, the sexual compulsivity scale is highly explicit concerning sexual activity and it is possible that some of the subjects were reluctant to disclose details on their intimate sexual lives. Thirdly, the participants were recruited using the “snow-ball” method in Internet forums of the gay and lesbian communities and we have no indication how reliable this may be.

## CONCLUSIONS

This study found associations between sexual compulsivity and attachment styles, which is a novel finding in an area of research that has not been thoroughly explored. Secondly, the finding of differences in sexual compulsivity between the homosexual and heterosexual populations merit further studies using larger populations.
